# Relationship between asthma and severe COVID-19: a national cohort study

**DOI:** 10.1136/thoraxjnl-2021-218629

**Published:** 2022-03-30

**Authors:** Ted Dolby, Vahe Nafilyan, Ann Morgan, Constantinos Kallis, Aziz Sheikh, Jennifer K Quint

**Affiliations:** 1 Office for National Statistics, Newport, UK; 2 National Heart and Lung Institute, Imperial College London, London, UK; 3 Usher Institute of Population Health Sciences and Informatics, University of Edinburgh, Edinburgh, UK

**Keywords:** asthma, COVID-19

## Abstract

**Background:**

We aimed to determine whether children and adults with poorly controlled or more severe asthma have greater risk of hospitalisation and/or death from COVID-19.

**Methods:**

We used individual-level data from the Office for National Statistics Public Health Data Asset, based on the 2011 census in England, and the General Practice Extraction Service data for pandemic planning and research linked to death registration records and Hospital Episode Statistics admission data. Adults were followed from 1 January 2020 to 30 September 2021 for hospitalisation or death from COVID-19. For children, only hospitalisation was included.

**Results:**

Our cohort comprised 35 202 533 adults and 2 996 503 children aged 12–17 years. After controlling for sociodemographic factors, pre-existing health conditions and vaccine status, the risk of death involving COVID-19 for adults with asthma prescribed low dose inhaled corticosteroids (ICS) was not significantly different from those without asthma. Adults with asthma prescribed medium and high dosage ICS had an elevated risk of COVID-19 death; HRs 1.18 (95% CI 1.14 to 1.23) and 1.36 (95% CI 1.28 to 1.44), respectively. A similar pattern was observed for COVID-19 hospitalisation; fully adjusted HRs 1.53 (95% CI 1.50 to 1.56) and 1.52 (95% CI 1.46 to 1.56) for adults with asthma prescribed medium and high-dosage ICS, respectively. Risk of hospitalisation was greater for children with asthma prescribed one (2.58 (95% CI 1.82 to 3.66)) or two or more (3.80 (95% CI 2.41 to 5.95)) courses of oral corticosteroids in the year prior to the pandemic.

**Discussion:**

People with mild and/or well-controlled asthma are neither at significantly increased risk of hospitalisation with nor more likely to die from COVID-19 than adults without asthma.

**Key messages**

**What is the key question?**

- It is not clear if children or adults with asthma are at greater risk of hospitalisation and/or death from COVID-19 compared with the general population.

**What is the bottom line?**

- Adults and children with poorly controlled or severe asthma are at significantly increased risk of hospitalisation from COVID-19 when compared with those without asthma, however, those with mild or well-controlled asthma are not.

**Why read on?**

- As the pandemic has progressed and more data has been gathered, it has become increasingly evident, at least as far as people with asthma are concerned, that the risks are not equal and perhaps the time has come to adopt more tailored strategies when developing guidelines and recommendations for vaccination policies aimed at protecting vulnerable populations from the risks of COVID-19.

## Introduction

As part of the response to the COVID-19 pandemic, a number of studies have been undertaken to ascertain who is most at risk of developing severe disease, and who therefore should be prioritised for vaccination and subsequent boosters.^1 2^ Asthma has been identified as one of several diseases associated with an increased risk of poorer COVID-19 outcomes, which has led to calls for people with asthma to be prioritised for vaccination and anti-viral/monoclonal antibodies.^3^

The picture that has emerged from the evidence so far is not entirely clear. Although asthma is not currently in the top 10 comorbidities associated with death in people with COVID-19, it has been suggested that asthma may increase the risk of developing more serious disease and thus hospitalisation for COVID-19.^1^ However, a recently published systematic review concluded that people with asthma are not at increased risk for acquiring COVID-19 compared with those without and also have similar outcomes following a COVID-19 infection compared with people without asthma.^4^ In addition, some studies have suggested that the use of inhaled corticosteroids (ICS) in asthma may have a protective effect and may be contributing to a decreased risk of severe COVID-19 disease.^5 6^

The current evidence base has some notable limitations. To date, most studies have been conducted in populations hospitalised for COVID-19 rather than people who contract COVID-19 in the community, or during the first wave of the pandemic and thus likely to be subject to selection biases. In addition, most studies have been conducted in adults rather than in children and with relatively short follow-up times. Another important limitation is that most studies do not differentiate mild asthma from more severe asthma or between poorly and well-controlled asthma, while outcomes may differ between these groups.^7^

In this study, we investigated the effect of asthma on the risk of COVID-19 hospitalisation and death using a unique linked administrative dataset for an exceptionally large cohort of adults and children over the age of 12 years living in England. We compared COVID-19 outcomes (hospitalisation and death) in people with and without asthma, and different severity of asthma. Our study aimed to answer the question as to whether children and adults with poorly controlled asthma have greater risk of hospitalisation and or death from COVID-19 and should be prioritised for vaccination or other interventions. In doing so, we aimed to bring some clarity to the seemingly conflicting/inconsistent evidence base to help policy-makers going forward.

## Methods

### Data sources

We used individual-level data from the Office for National Statistics (ONS) Public Health Data Asset, a unique cohort based on the 2011 census in England, and the General Practice Extraction Service (GPES) data for pandemic planning and research which are linked to death registration records and Hospital Episode Statistics (HES) hospital admission data. The ONS 2011 Census data were linked to the 2011–2013 NHS Patient Registers in order to obtain NHS numbers for each individual. Data were first linked deterministically using 24 different matching keys, based on a combination of forename, surname, date of birth, sex and geography (postcode or Unique Property Reference Number). Probabilistic matching was then used to attempt to match records that were not linked deterministically, using 13 different combinations of personal identifiers. Candidate matches were assigned to census records using the Felligi-Sunter probabilistic matching method. Of the 53 483 502 census records, 50 019 451 were linked deterministically. 555 291 additional matches were obtained using probabilistic matching (overall linkage rate: 94.6%).

We restricted our sample to individuals who were aged 12 or over at the beginning of the pandemic. Of the 51 418 746 people enumerated at the 2011 Census in England and Wales, we excluded 2 832 087 people (5.5%) who could not be linked deterministically or probabilistically to the NHS Patient register, and 4 032 701 individuals (7.8%) who had died between the 2011 Census and 1 January 2020. An additional 6 350 661 people (12.4%) were not linked to the English primary care records, either because they did not live in England in 2019 (the census included people living in England and Wales), or because they were not registered with the NHS and were also excluded. After excluding 4261 individuals who had received their vaccine before the beginning of the vaccination campaign (8 December 2020), our sample consisted of 38 199 036 individuals (sample flow diagram online supplemental table S1).

10.1136/thoraxjnl-2021-218629.supp1 Supplementary data

Our final cohort comprised 35 202 533 adults (18+) and 2 996 503 children aged 12–17, corresponding to 79.2% and 76.5% of the total respective populations in England (based on the 2020 mid-year population estimates).^8^

### Outcomes

Individuals in the study population were followed up from 1 January 2020 to 30 September 2021 for COVID-19 death, defined as a death where COVID-19 was recorded as an underlying or contributory cause of death on the death certificate as identified by two International Classification of Diseases and Related Health Problems (ICD-10 codes U07.1 (confirmed) or U07.2 (suspected). The secondary outcome was hospitalisation with COVID-19 between 1 January 2020 and 30 September 2021, defined as ICD-10 codes U07.1 (confirmed) or U07.2 (suspected). For children, we only included hospitalisations, as the number of deaths was too low to conduct a meaningful statistical analysis.

### Exposures

Our control group was those without an asthma diagnosis the 2 years prior to the pandemic (1 January 2018 to 31 December 2019) and our exposure groups were: (1) asthma diagnosis and no ICS prescriptions in the year prior to the pandemic (1 January 2019 to 31 December 2019); (2) asthma diagnosis and low dosage ICS prescribed in the year prior to the pandemic (3) asthma diagnosis and medium dosage ICS prescribed in the year prior to the pandemic; and (4) asthma diagnosis and high-dosage ICS prescribed in the year prior to the pandemic. We also used oral corticosteroids (OCS) prescriptions as an exposure variable (as a measure of exacerbation frequency and thus poor control). For this, our control group was again no asthma diagnosis, and our exposure categories were: (1) asthma diagnosis and no OCS prescription in the year prior to the pandemic (1 January 2019 to 31 December 2019); (2) asthma diagnosis and one OCS prescription in the year prior to the pandemic and (3) asthma diagnosis and two or more OCS prescriptions in the year prior to the pandemic.

Asthma diagnosis was defined by the presence of at least one relevant SNOMED code in the primary care record in the 2 years prior to the pandemic (1 January 2018 to 1 January 2020). The asthma definition has been validated for adults in a previous study using the Clinical Practice Research Datalink (CPRD) GOLD database against a reference standard of physician review of patient notes and has a high positive predictive value (>86%).^9^ Prescriptions for ICS and OCS in the 1 year prior to the pandemic (1 January 2019 to 31 December 2019) were used to define the exposure categories. In assigning individuals to low, medium or high ICS dosage level, highest dosage the individual was prescribed during this baseline period was used. For adults, OCS prescriptions were counted if they were below 300 mg total dose. The SNOMED codes used for ICS and OCS prescriptions were based on previous work, along with low/medium/high classifications for ICS and exacerbation levels for OCS and based on the National Institute for Health and Care Excellence guidelines, taking both the active ingredient and inhaler type into account.^10^

We did not add any additional restrictions to determine whether the OCS prescription was for asthma or not, we simply required the individual to have received an asthma diagnosis in the previous 2 years and have received an OCS prescription under 300 mg for selected SNOMED codes. Given that acute exacerbations of asthma in adults and children over 12 are routinely treated with OCS (prednisolone) at a dosage level of 40–50 mg once a day for 5 days, OCS prescriptions were only counted if they were below 300 mg (total dose). This methodology has been used in previous studies.^11^

### Covariates

We adjusted for factors that could confound the relationship between asthma and COVID-19 hospitalisation or death. We adjusted for sex, age, region, ethnicity (10-categories), quintile of the Index of Multiple Deprivation (IMD) and several relevant pre-existing conditions, defined as per in the QCovid risk model (online supplemental table S2).^6^ We also used hospitalisation in the year before the pandemic for any cause other than asthma as a measure of underlying health. We also adjusted for the number of OCS prescriptions under 300 mg in the year prior to the pandemic and COVID-19 vaccination status, defined as having received one, two or no doses of a COVID-19 vaccine, and allowing the status to vary over time.

### Statistical analyses

As a measure of differences in absolute risk of COVID-19 hospitalisation and death between people with asthma and the general population, we calculated age-standardised rates, whereby the age distribution within each group was standardised to the 2013 European Standardised Population. We calculated age standardised rates separately for men and women for adults (18 or over). For children (12–17), we calculated crude rates.

To estimate the relative risk of COVID-19 mortality between people with a diagnosis of asthma and the general population, we fitted Cox proportional hazard models, adjusted for a range of potential confounding factors, using separate models for adults and children. We fitted several models, adjusting for confounding factors sequentially to assess how they affected the hazard ratios of having asthma. We used different model specifications for children and for adults. For adults, the first model was just adjusted for age and sex. Second, we further adjusted for socio-demographic factors, including region, ethnicity, and quintile of IMD. Region was adjusted for by estimating region-specific baseline hazard, to capture changes in infection rate over time in different areas. Third, we added comorbidities, hospitalisation in the previous year for any other reason than asthma, and OCS use except when OCS was the exposure variable. Finally, we added vaccination status as a time varying covariate. For children, the first two models were the same as for adults. However, in the third model we adjusted for hospitalisation in the previous year for any other reason than asthma.

## Results

### Characteristics of the study population

Our analytical sample consisted of 35 202 533 adults (aged ≥18 year) and 2 996 503 children aged 12–17 years who were alive on 1 January 2020 and living in England in private households. Over the period, there were 118 256 (0.3%) deaths involving COVID-19 and 331 063 (0.9%) COVID-19 hospitalisations in adults (table 1). In children (12–17), there were 21 (<0.01%) deaths involving COVID-19 and 2930 (0.1%) hospitalisations. Among adults, 2 671 931 (7.6%) had asthma (defined as an asthma diagnostic code in the prior 2 years), with 1 451 443 (54.3%) prescribed low dose ICS, 519 294 (19.4%) medium dosage ICS, and 136 080 (5.1%) high dosage ICS; 307 486 (11.5%) had one and 291 970 (10.9%) at least two OCS prescriptions in the baseline period. In children, 215 873 (7.2%) had an asthma diagnosis in the 2 years prior.

**Table 1:** Baseline characteristics of adults and children included in the cohort

		Adults		Children	
		N	%	N	%
Death involving COVID-19		118 256	0.3	21	<0.01
COVID-19 hospitalisation		331 063	0.9	2930	0.1
ICS	No asthma	32 530 602	92.4		
	No ICS	565 114	1.6		
	Low ICS	1 451 443	4.1		
	Medium ICS	519 294	1.5		
	High ICS	136 080	0.4		
OCS	No asthma	32 530 602	92.4	2 780 630	92.8
	0 OCS	2 072 475	5.9	197 867	6.6
	1 OCS	307 486	0.9	12 944	0.4
	2+ OCS	291 970	0.8	5062	0.2
Sex	Female	18 448 204	52.4	1 470 953	49.1
	Male	16 754 329	47.6	1 525 550	50.9
Age	Mean		51.0		14.9
	SD		19.0		1.7
Ethnic group	Bangladeshi	263 624	0.7	48 062	1.6
	Black African	528 677	1.5	84 971	2.8
	Black Caribbean	378 314	1.1	26 803	0.9
	Chinese	186 841	0.5	12 125	0.4
	Indian	939 958	2.7	86 380	2.9
	Mixed	566 176	1.6	150 846	5.0
	Other	846 287	2.4	109 042	3.6
	Pakistani	693 045	2.0	120 282	4.0
	White British	29 074 171	82.6	2 245 850	74.9
	White other	1 725 440	4.9	112 142	3.7
Quintile of Index of Multiple Deprivation	1	6 478 687	18.4	662 546	22.1
	2	6 874 247	19.5	582 881	19.5
	3	7 189 513	20.4	562 070	18.8
	4	7 324 784	20.8	571 206	19.1
	5	7 335 302	20.8	617 800	20.6
Region	North East	1 767 939	5.0	138 832	4.6
	North West	4 723 007	13.4	396 101	13.2
	Yorkshire and The Humber	3 593 129	10.2	304 752	10.2
	East Midlands	3 141 826	8.9	260 331	8.7
	West Midlands	3 718 733	10.6	321 909	10.7
	East of England	4 008 795	11.4	344 332	11.5
	London	4 826 372	13.7	444 266	14.8
	South East	5 770 900	16.4	498 722	16.6
	South West	3 651 832	10.4	287 258	9.6
Hospitalised in previous year (excluding for asthma)		5 639 375	16.0	160 463	5.4
Learning disability	None	34 862 723	99.0		
	Other learning disabilities	332 557	0.9		
	Down’s syndrome	7253	0.02		
Chronic kidney disease	None	34 697 556	98.6		
	CDK 3	436 452	1.2		
	CDK 4	45 653	0.1		
	CDK 5	22 872	0.1		
Other conditions	Diabetes	2 558 503	7.3		
	COPD	915 698	2.6		
	Stroke	513 584	1.5		
	Atrial fibrillation	797 049	2.3		
	Congestive cardiac failure	410 692	1.2		
	Venous thromboembolism	9012	0.03		
	Peripheral vascular disease	166 909	0.5		
	Dementia	371 454	1.1		
	Parkinson’s disease	91 259	0.3		
	Epilepsy	192 571	0.5		
	Severe mental illness	3 249 258	9.2		
	Osteoporotic fracture	18 891	0.1		
	Rheumatoid arthritis or systemic lupus erythematosus	239 841	0.7		
	Cirrhosis of the liver	54 585	0.2		

CKD, chronic kidney disease; COPD, chronic obstructive pulmonary disease; ICS, inhaled corticosteroids; OCS, oral corticosteroids.

### Age-standardised mortality and hospitalisation rates

Table 2 shows the age-standardised mortality and hospitalisation rates for COVID-19 by asthma diagnosis separately for children and adults. Adults (18+) with asthma prescribed medium or high dose ICS had an elevated risk of COVID-19 death compared with people without asthma, with age-standardised mortality rates (ASMR) of 439.8 (95% confidence intervals 424.1–455.5) and 554.9 (521.2–588.9) per 100 000 people, respectively. By contrast, the ASMR for adults without asthma was 304.4 (302.6–306.2) and 317.4 (308.6–326.2) for people with asthma prescribed a low ICS. The differences in ASMRs were more pronounced among younger people (18–39 and 40–49) than in older people (50 years old or over) (online supplemental table S3). While the ASMRs were lower in women than men, the relative differences were larger in women than men (online supplemental table S4). Similar patterns were observed for OCS prescriptions, with adults prescribed OCS having an elevated risk of death. Similar patterns were observed for age-standardised hospitalisation rates in adults.

**Table 2:** Age-standardised COVID-19 mortality and hospitalisation rates per 100 000 population, stratified by asthma status

Exposure	Deaths involving COVID-19	COVID-19 hospitalisation	Population	Age-standardised mortality rates per 100 000	Age-standardised hospitalisation rates per 100 000
Adults (18+), ICS					
No asthma	106 636	291 685	32 530 602	304.4 (302.6 to 306.2)	857.3 (854.1 to 860.4)
No ICS	2052	6086	565 114	408.8 (391.0 to 426.6)	1166.3 (1136.8 to 1195.8)
Low ICS	5211	18 689	1 451 443	317.4 (308.6 to 326.2)	1175.4 (1158.2 to 1192.6)
Medium ICS	3201	10 907	519 294	439.8 (424.1 to 455.5)	1729.2 (1693.8 to 1764.6)
High ICS	1156	3696	136 080	554.9 (521.2 to 588.6)	2074.4 (1999.4 to 2149.4)
Adults (18+), OCS					
No asthma	106 636	291 685	32 530 602	304.4 (302.6 to 306.2)	857.3 (854.1 to 860.4)
0 OCS	7146	24 898	2 072 475	322.0 (314.5 to 329.6)	1132.9 (1118.6 to 1147.1)
1 OCS	1519	5457	307 486	399.4 (378.9 to 419.9)	1570.6 (1527.2 to 1613.9)
2+OCS	2955	9023	291 970	624.2 (600.2 to 648.3)	2369.0 (2312.3 to 2425.7)
Children (12-17), OCS		COVID-19 hospitalisation	Population		Hospitalisation rates per 100 000
No asthma	–	2625	2 780 630	–	94.4 (90.8 to 98.0)
0 OCS	–	254	197 867	–	128.4 (112.6 to 144.1)
1 OCS	–	32	12 944	–	247.2 (161.7 to 332.8)
2+ OCS	–	19	5062	–	375.3 (206.9 to 543.8)

The rates were standardised to the 2013 European Standardised population. 95% CIs of the age standardised rates in brackets.

ICS, inhaled corticosteroids; OCS, oral corticosteroids.

Children with asthma had higher risk of COVID-19 hospitalisation over the period of study. The rate of COVID-19 hospitalisation in children with poorly controlled asthma (two or more courses of OCS) was 375.3 (206.9–543.8) per 100 000 children, compared with 94.4 (90.8–98.0) for children without asthma. COVID-19 hospitalisation rates were also elevated in children with a diagnosis of asthma who were not prescribed OCS, but to a lesser extent, with hospitalisation rates of 128.4 (112.6–144.1).

### Adjusted HRs

**Figure 1:**
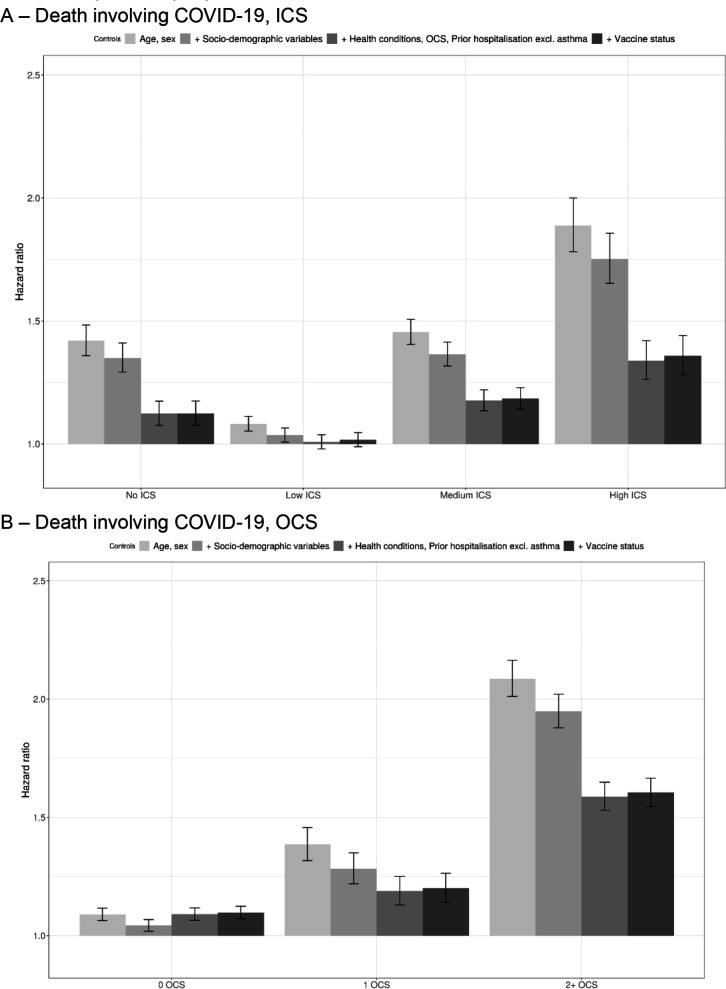
Adjusted HRs of death involving COVID-19 in adults for different asthma status compared with people with no asthma. HRs of death involving COVID-19 compared with people with no asthma, obtained from Cox regression models. Sociodemographic factors include, region, ethnicity, quintile of the Index of Multiple Deprivation; health conditions include relevant pre-existing conditions, defined as per in the QCovid risk model (see online supplemental table S2 for details on the variables used in this analysis). ICS, inhaled corticosteroids; OCS, oral corticosteroids.

Figure 1 reports adjusted hazard ratios of death involving COVID-19 in adults for different asthma exposure categories compared with people without asthma. After controlling for sociodemographic factors, pre-existing health conditions and vaccine status, the risk of death involving COVID-19 for adults with asthma prescribed low-dose ICS was not significantly different from those without asthma (figure 1A). However, adults with asthma prescribed medium and high dosage ICS had an elevated risk of COVID-19 death, with HRs of 1.18 (95% CI 1.14 to 1.23) and 1.36 (95% CI 1.28 to 1.44), respectively. A similar pattern was observed when examining COVID-19 hospitalisation, but the relative risks were slightly higher, with fully adjusted HRs of 1.53 (95% CI 1.50 to 1.56) and 1.52 (95% CI 1.46 to 1.56) for adults with asthma prescribed medium and high dosage ICS, respectively (online supplemental table S4).

Similarly, adults with asthma prescribed two or more courses of OCS in the year prior to the pandemic had a notably higher risk of death than those without asthma, with a HR of 1.60 (95% CI 1.55 to 1.67) (figure 1B). The relative risk was lower for those with asthma prescribed zero (1.10 (95% CI 1.07 to 1.12)) or one (1.20 (95% CI 1.14 to 1.26)) course of OCS but remained significantly greater than 1. HRs for COVID-19 hospitalisation showed the same pattern, but were slightly higher, with adults with asthma prescribed two or more courses of OCS having an HR of COVID-19 hospitalisation of 1.94 (95% CI 1.90 to 1.99). For both ICS and OCS, adjusting for pre-existing health conditions had a strong effect on the effect estimates, whereas adjustment for sociodemographic factors or vaccination status did not substantially affect the results.

**Figure 2:**
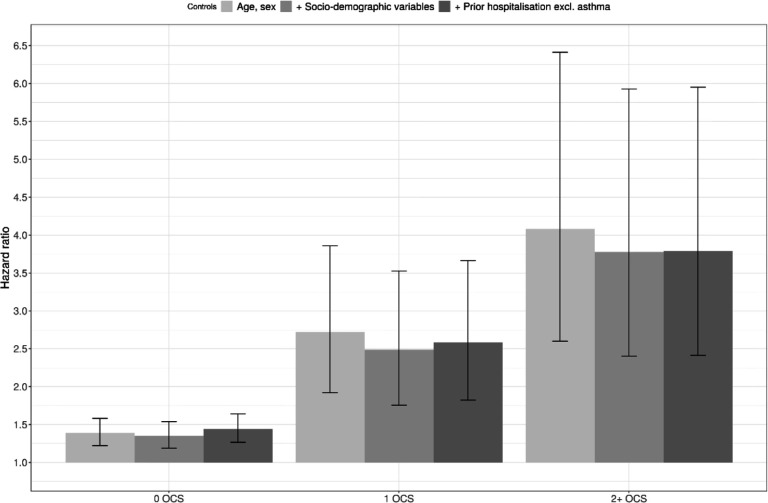
Adjusted HRs of COVID-19 hospitalisation in children for different asthma status compared with children with no asthma. HRs of death COVID-19 hospitalisation compared with people with no asthma, obtained from Cox regression models. Sociodemographic factors include, region, ethnicity, quintile of the Index of Multiple Deprivation (see online supplemental table S2 for details on the variables used inthis analysis). OCS, oral corticosteroids.

Figure 2 reports adjusted HRs of COVID-19 hospitalisation in children aged 12–17 years for different asthma exposure groups compared with children without asthma. The risk of hospitalisation was greater for children with asthma than those without asthma. This risk was greater for those children with asthma prescribed one (2.58 (95% CI 1.82 to 3.66)) or two or more (3.80 (95% CI 2.41 to 5.95)) courses of OCS in the year prior to the pandemic.

Examining adjusted HRs stratified by sex and age group, we found that the relative risks tended to be greater in the groups with lower absolute risk (online supplemental table S4). The adjusted HRs of hospitalisation and death were greater for females with asthma than males with asthma in both ICS or OCS exposure groups compared with people without asthma. Similarly, the adjusted HRs of hospitalisation for adults with asthma were greater among younger age groups (18–39 years old and 40–49 years old), particularly for adults who had been prescribed ICS or OCS in the year prior to the pandemic. There was no statistically significant difference in the relative risk of hospitalisation or death for adults with asthma in both ICS or OCS exposure groups between the vaccinated and unvaccinated groups (online supplemental table S4).

## Discussion

### Main findings

Our study is one of the most comprehensive, and certainly the largest, longitudinal cohort study to investigate COVID-19 hospitalisation and mortality in people with current asthma that has been conducted to date. We found that adults with mild and/or well-controlled asthma are neither at significantly increased risk of being hospitalised with COVID-19 nor more likely to die from COVID-19 than adults without asthma. However, those who recently required higher ICS dosage-based therapies (ie, have more severe disease) or who experience one or more exacerbations per year (ie, have poorly-controlled asthma) as measured by OCS prescriptions, are potentially at increased risk of both hospitalisation and death. Specifically, we found that two or more courses of OCS in the preceding year was associated with an increase in hospitalisation in both children and adults and associated with death in adults.

We also found that there was no difference in COVID-19 hospitalisation and death between adults with asthma prescribed low-dose ICS and those with no history of asthma, whereas there was a slightly increased risk in those with a diagnosis of asthma but not prescribed ICS, again suggesting that better control of asthma reduces relative risk of adverse COVID-19 outcomes. Finally, whereas our analysis indicates that the relative risks of COVID-19 hospitalisation and death are higher in women than men, and in younger as opposed to older age groups (ie, 18–39 years relative to 50 years and over), it is important to note that the absolute risks were greater in men and in the over 50 s.

### Comparison with other studies

Several researchers have alluded to the apparent inconsistencies in the literature regarding the vulnerability of people with asthma to COVID-19. In this context, it is noteworthy that many of the earlier studies that have suggested that people with asthma are at increased risk of severe COVID-19 were conducted in the first 6–9 months of the pandemic and predominantly in hospitalised patients.^12^ It is also worth noting that among this group of studies, several also reported that the association is stronger among those with non-allergic asthma, whereas risks among those with allergic asthma were either only slightly elevated (relative to the general population) or statistically non-significant.^13^

In contrast, later studies, including several conducted in community cohorts and therefore more representative of the asthma population, have failed to find strong evidence of an association between asthma and increased risk of poor outcomes, including hospitalisation, ICU admission and death from COVID-19.^14–18^ For example, according to Doherty *et al*, while 14% of people admitted to hospital for COVID-19 (n=20 133) had asthma, a prior diagnosis of asthma was not a significant risk factor for COVID-19 mortality; however, prior diagnosis of other chronic diseases including chronic cardiac disease, chronic non-asthmatic pulmonary disease and chronic kidney disease were independently associated with in-hospital COVID-19 mortality.^14^ A Spanish community-based study of over 70 000 people with asthma also showed that severe COVID-19 disease was associated, in addition to age and sex, with the presence of multiple comorbidities, in particular obesity, hypertension, dyslipidaemia and diabetes. In our study, we found that the addition of comorbidities to our Cox regression models produced a sizeable attenuation in the relative risks for poor COVID-19 outcomes, adding further weight to the implication from other studies that age and comorbidities other than asthma are what is really driving the worse outcomes.^19^

While, in common with other studies, we found that people with mild and/or well-controlled asthma were not necessarily at greater risk of COVID-19 hospitalisation and death than people without asthma we did see an increased risk among those with more severe asthma (prescribed ICS at medium or high doses as opposed to low) and whose asthma is less well controlled, determined by OCS prescriptions in the year prior. Our findings in this regard build on those from other studies, which have not had the advantage of being able to grade disease severity in terms of ICS dosage level but have instead used proxies—either number of inhaled treatments or recent OCS use or hospitalisation—to categorise people according to their disease severity.^20 21^ For instance, severe asthma, defined as recent OCS use, was one of several factors found to be associated with COVID-19 mortality in the UK OpenSAFELY cohort of patients hospitalised with COVID-19.^1^ A second OpenSAFELY study showed that prescription of high-dose ICS was associated with a 55% increased risk of death from COVID-19 and concluded this was likely due to disease severity.^22^ Another UK study also concluded that people with more severe asthma, in this case defined by number of ICS prescriptions in 1 year, were at increased of risk of more serious COVID-19 outcomes than those with milder disease.^23^ While the prior evidence for the association with disease severity is somewhat muddied by difficulties in differentiating disease severity from disease control, it is more robust for the association with disease control. As in our study, several studies, including one from the Republic of Korea, have shown that a history of acute exacerbation is significant risk factor for death among COVID-19 patients with asthma, a finding which underscores the importance of good control in the management of asthma.^12^

This study goes some way towards resolving some of the apparent inconsistencies in the literature surrounding asthma and COVID-19 which to a certain extent has been inevitable given the pace of the pandemic and urgent need for information on which to base decisions about vaccination priorities, among other interventions. Studies to date have been challenging to interpret into policy decisions due to a combination of factors, including limited cohort sizes, differing comparator arms used in studies, less granular disease management definitions, shorter follow-up, selected cohorts, not necessarily taking early shielding into account and most studies limited to hospitalised populations and the first wave of pandemic data when testing was more limited. All of these issues may generate biased results.

### Strengths and limitations

The primary strength of our study is the use of a uniquely large, nationwide population-level dataset, created for pandemic planning and research purposes. This dataset is based on GPES (GPDPR, general practice data for planning and research) Data linked to the most recent census data, mortality records and HES. Our data cover around 80% of the population of England, and includes children aged 12 and over; however, we have not explored this question in children under the age of 12. By combining electronic healthcare records with self-reported data from the detailed 2011 census means we have been able to measure some key sociodemographic confounders with a high of accuracy. For instance, we used self-reported ethnicity from census data rather than ethnicity recorded in primary care or hospitalisation data, which is of lower quality and suffers from a high degree of missingness. The granularity of prescription information which has allowed us to tease out poor control from disease severity represents another major strength of our study. The main limitation is that the study population is limited to people enumerated at the 2011 census, and therefore did not include people who have since immigrated or were born between 2011 and 2020. As a result, it did not fully represent the population at risk. However, migrants tend to be young and the risk of COVID-19 mortality is low for young people. Finally, there are inevitably issues of misclassification with any study using routine electronic healthcare records. Where possible we have used validated definitions, and the team have many years of experience in using these data.

### Policy implications

In a rapidly evolving pandemic, keeping pace with the volume of new data and evidence is challenging. It also makes it difficult to provide straightforward answers to some of the key important policy questions, such as are people with asthma at a higher risk of poorer clinical outcomes from COVID-19? As the pandemic has progressed and more data has been gathered, it has become increasingly evident, at least as far as people with asthma are concerned, that the risks are not equal in that not all asthma is the same; those with mild/well-controlled asthma have a similar risk to the general population. It is only those with poorly controlled or severe disease that are at increased risk of serious COVID-19 adverse outcomes and may need prioritisation for vaccination and/or other treatment. Perhaps the time has come to adopt more tailored strategies when developing guidelines and recommendations for vaccination policies aimed at protecting vulnerable populations from the risks of COVID-19. It also important when planning public health policy and interventions not to focus on individual diseases, but to contextualise the strength of association between asthma risk and outcomes considering other chronic diseases. Whatever the risks to any one individual, the importance of disease control at all levels of disease severity is a consistent message and serves as a reminder that even in a time of COVID-19 the basics of routine healthcare should not be overlooked.

## Conclusion

Good disease management including treatment adherence leading to better disease control helps to limit the risk of poor outcomes. In a rapidly evolving pandemic, as information develops, messaging changes and when contextualising disease outcomes, it is important to consider relative and absolute risks.

## Data Availability

Data may be obtained from a third party and are not publicly available.
