# Supplementary material for: Relationship between asthma and severe COVID-19: a national cohort study
Source: Thorax. 2022 Mar 30:thoraxjnl-2021-218629. doi: 10.1136/thoraxjnl-2021-218629 (PMC8983409; doi:10.1136/thoraxjnl-2021-218629)
Supplement: Supplementary file 1 [file thoraxjnl-2021-218629supp001.pdf]

Supplement

Table S1 Sample selection and number of participants

Inclusion criteria	Cohort size	Proportion (%)
All enumerated at 2011 Census in England and Wales, aged 12+ on 1 January 2020	51,418,746	
Linked to the NHS 2011-2013 Patient Register	48,586,659	94.5
Alive on 1 January 2020	44,553,958	86.6
Living in England and linked to 2019 English primary care records	38,203,297	74.3
Received vaccination after the beginning of the vaccination campaign (8 December 2020)	38,199,036	74.3

Table S2: Variables used in the analyses

Variable	Coding	Source
**Outcomes**		
COVID-19 death	Confirmed (ICD -10 codes U07.1) or suspected (U07.2) as underlying or contributory cause of death on the death certificate	2020 - 2021 Death registration
COVID-19 hospitalisation	Confirmed (ICD -10 codes U07.1) or suspected (U07.2) as primary diagnosis	2020 - 2021 HES
**Exposure**		
ICS	No asthma diagnosis in 2018-2019, Asthma diagnosis in 2018-2019 and no inhaled corticosteroid (ICS) prescribed in 2019, Asthma diagnosis in 2018-2019 and low dosage ICS prescribed in 2019, Asthma diagnosis in 2018-2019 and medium dosage ICS prescribed in 2019, Asthma diagnosis in 2018-2019 and high dosage ICS prescribed in 2019	GDPPR
OCS	No asthma diagnosis in 2018-2019, Asthma diagnosis in 2018-2019 and no oral corticosteroid (OCS) prescribed in 2019, Asthma diagnosis in 2018-2019 and one OCS prescription in 2019, Asthma diagnosis in 2018-2019 and two or more OCS prescriptions in 2019	GDPPR
**Confounding factors**		
Age on 1 January 2020	Third-order polynomial	2011 Census
Sex	Female, Male	2011 Census

Self-reported ethnic group	Bangladeshi, Black African, Black Caribbean, Chinese, Indian, Mixed, Pakistani, White British, White Other, Other	2011 Census
Region of residence in 2019	Specific baseline hazard for each Government Office region (East Midlands, East of England, London, North East, North West, South East, South West, West Midlands, Yorkshire and the Humber)	GDPPR
Index of Multiple Deprivation (IMD)	Binary variables representing quintiles of the index of multiple deprivation (Decile 1: least deprived; Decile 5: most deprived)	GDPPR
Recent hospitalisation	Any hospitalisation in 2019 for reasons other than asthma	2019 HES
Comorbidities	Chronic Kidney Disease (No CKD, CKD3, CKD4, CKD5), Learning disability (No learning disability, Down's Syndrome, other learning disability); binary variables for Diabetes, Chronic obstructive pulmonary disease (COPD), Stroke , Atrial fibrillation , Congestive cardiac failure , Venous thromboembolism , Peripheral vascular disease, Dementia , Parkinson's disease , Epilepsy , Severe mental illness (bipolar disorder, schizophrenia, severe depression), Osteoporotic fracture, Rheumatoid arthritis or Systemic lupus erythematosus, and Cirrhosis of the liver	2015-2019 GDPPR, HES

Vaccination status	Not vaccinated; 1 dose, 2 doses; Vaccination status varies over time	NIMS
OCS use	Number of prescriptions of oral corticosteroids in 2019 (0 prescriptions, 1 prescription, 2 or more prescriptions)	GDPPR

Note: Comorbidities were defined using the same SNOMED codes and ICD-19 codes as for the QCovid risk model [6].

Table S3 Age-standardised COVID-19 mortality and hospitalisation rates, stratified by asthma status, age group, sex and vaccination status

Outcome	Exposure	Exposure group	Age group	Sex	Event	Population	Rate	Age-standardised rate
Death involving COVID-19	ICS	No asthma	Adults	All	106,636	32,530,602	327.8 [325.8-329.8]	304.4 [302.6-306.2]
Death involving COVID-19	ICS	No ICS	Adults	All	2,052	565,114	363.1 [347.4-378.8]	408.8 [391.0-426.6]
Death involving COVID-19	ICS	Low ICS	Adults	All	5,211	1,451,443	359.0 [349.3-368.8]	317.4 [308.6-326.2]
Death involving COVID-19	ICS	Medium ICS	Adults	All	3,201	519,294	616.4 [595.1-637.7]	439.8 [424.1-455.5]
Death involving COVID-19	ICS	High ICS	Adults	All	1,156	136,080	849.5 [800.7-898.3]	554.9 [521.2-588.6]
Death involving COVID-19	ICS	No asthma	Adults	Female	47,991	16,848,059	284.8 [282.3-287.4]	237.4 [235.3-239.5]
Death involving COVID-19	ICS	No ICS	Adults	Female	1,084	327,992	330.5 [310.9-350.1]	349.9 [328.9-370.8]
Death involving COVID-19	ICS	Low ICS	Adults	Female	2,868	871,384	329.1 [317.1-341.2]	277.4 [267.2-287.7]
Death involving COVID-19	ICS	Medium ICS	Adults	Female	1,816	318,711	569.8 [543.7-595.9]	398.1 [379.3-416.9]
Death involving COVID-19	ICS	High ICS	Adults	Female	609	82,058	742.2 [683.4-800.9]	496.1 [454.7-537.5]
Death involving COVID-19	ICS	No asthma	Adults	Male	58,645	15,682,543	374.0 [370.9-377.0]	389.2 [386.0-392.4]
Death involving COVID-19	ICS	No ICS	Adults	Male	968	237,122	408.2 [382.6-433.9]	509.5 [476.7-542.3]
Death involving COVID-19	ICS	Low ICS	Adults	Male	2,343	580,059	403.9 [387.6-420.2]	389.4 [372.9-405.8]
Death involving COVID-19	ICS	Medium ICS	Adults	Male	1,385	200,583	690.5 [654.2-726.7]	512.2 [484.0-540.5]
Death involving COVID-19	ICS	High ICS	Adults	Male	547	54,022	1012.6 [928.1-1097.0]	640.7 [583.5-697.8]
Death involving COVID-19	ICS	No asthma	18-39	All	704	10,625,503	6.6 [6.1-7.1]	6.8 [6.3-7.3]
Death involving COVID-19	ICS	No ICS	18-39	All	23	221,307	10.4 [6.1-14.6]	11.3 [7.1-17.0]
Death involving COVID-19	ICS	Low ICS	18-39	All	47	373,650	12.6 [9.0-16.2]	12.7 [9.3-16.9]
Death involving COVID-19	ICS	Medium ICS	18-39	All	31	87,936	35.3 [22.8-47.7]	31.5 [21.4-44.8]
Death involving COVID-19	ICS	High ICS	18-39	All	8	19,773	40.5 [12.4-68.5]	37.8 [16.1-74.8]
Death involving COVID-19	ICS	No asthma	40-49	All	1,535	5,214,124	29.4 [28.0-30.9]	29.0 [27.5-30.4]
Death involving COVID-19	ICS	No ICS	40-49	All	36	93,641	38.4 [25.9-51.0]	38.0 [26.6-52.6]
Death involving COVID-19	ICS	Low ICS	40-49	All	146	233,875	62.4 [52.3-72.5]	60.6 [50.7-70.5]
Death involving COVID-19	ICS	Medium ICS	40-49	All	83	77,770	106.7 [83.8-129.7]	103.0 [81.8-128.0]
Death involving COVID-19	ICS	High ICS	40-49	All	27	17,826	151.5 [94.4-208.6]	143.0 [93.6-209.0]
Death involving COVID-19	ICS	No asthma	50+	All	104,397	16,690,975	625.5 [621.7-629.3]	614.6 [610.9-618.4]
Death involving COVID-19	ICS	No ICS	50+	All	1,993	250,166	796.7 [761.8-831.5]	824.3 [787.9-860.6]
Death involving COVID-19	ICS	Low ICS	50+	All	5,018	843,918	594.6 [578.2-611.0]	626.0 [608.4-643.6]

Death involving COVID-19	ICS	Medium ICS	50+	All	3,087	353,588	873.0 [842.4-903.7]	850.7 [820.3-881.2]
Death involving COVID-19	ICS	High ICS	50+	All	1,121	98,481	1138.3 [1072.0-1204.5]	1070.0 [1005.9-1134.1]
Death involving COVID-19	OCS	No asthma	Adults	All	106,636	32,530,602	327.8 [325.8-329.8]	304.4 [302.6-306.2]
Death involving COVID-19	OCS	0 OCS	Adults	All	7,146	2,072,475	344.8 [336.8-352.8]	322.0 [314.5-329.6]
Death involving COVID-19	OCS	1 OCS	Adults	All	1,519	307,486	494.0 [469.2-518.8]	399.4 [378.9-419.9]
Death involving COVID-19	OCS	2+ OCS	Adults	All	2,955	291,970	1012.1 [975.8-1048.4]	624.2 [600.2-648.3]
Death involving COVID-19	OCS	No asthma	Adults	Female	47,991	16,848,059	284.8 [282.3-287.4]	237.4 [235.3-239.5]
Death involving COVID-19	OCS	0 OCS	Adults	Female	3,743	1,199,878	311.9 [302.0-321.9]	273.6 [264.7-282.4]
Death involving COVID-19	OCS	1 OCS	Adults	Female	877	202,025	434.1 [405.4-462.8]	353.4 [329.7-377.1]
Death involving COVID-19	OCS	2+ OCS	Adults	Female	1,757	198,242	886.3 [845.0-927.5]	572.3 [544.0-600.5]
Death involving COVID-19	OCS	No asthma	Adults	Male	58,645	15,682,543	374.0 [370.9-377.0]	389.2 [386.0-392.4]
Death involving COVID-19	OCS	0 OCS	Adults	Male	3,403	872,597	390.0 [376.9-403.1]	403.5 [389.4-417.5]
Death involving COVID-19	OCS	1 OCS	Adults	Male	642	105,461	608.8 [561.8-655.7]	489.1 [449.7-528.4]
Death involving COVID-19	OCS	2+ OCS	Adults	Male	1,198	93,728	1278.2 [1206.3-1350.1]	722.0 [676.9-767.2]
Death involving COVID-19	OCS	No asthma	18-39	All	704	10,625,503	6.6 [6.1-7.1]	6.8 [6.3-7.3]
Death involving COVID-19	OCS	0 OCS	18-39	All	74	592,647	12.5 [9.6-15.3]	12.9 [10.1-16.2]
Death involving COVID-19	OCS	1 OCS	18-39	All	9	68,195	13.2 [4.6-21.8]	12.4 [5.6-23.5]
Death involving COVID-19	OCS	2+ OCS	18-39	All	26	41,824	62.2 [38.3-86.1]	57.2 [37.1-84.2]
Death involving COVID-19	OCS	No asthma	40-49	All	1,535	5,214,124	29.4 [28.0-30.9]	29.0 [27.5-30.4]
Death involving COVID-19	OCS	0 OCS	40-49	All	178	338,439	52.6 [44.9-60.3]	51.2 [43.6-58.8]
Death involving COVID-19	OCS	1 OCS	40-49	All	48	48,571	98.8 [70.9-126.8]	94.7 [69.7-125.8]
Death involving COVID-19	OCS	2+ OCS	40-49	All	66	36,102	182.8 [138.7-226.9]	177.6 [136.9-226.6]
Death involving COVID-19	OCS	No asthma	50+	All	104,397	16,690,975	625.5 [621.7-629.3]	614.6 [610.9-618.4]
Death involving COVID-19	OCS	0 OCS	50+	All	6,894	1,141,389	604.0 [589.8-618.2]	638.8 [623.6-654.1]
Death involving COVID-19	OCS	1 OCS	50+	All	1,462	190,720	766.6 [727.4-805.7]	783.6 [742.8-824.5]
Death involving COVID-19	OCS	2+ OCS	50+	All	2,863	214,044	1337.6 [1288.9-1386.2]	1187.3 [1142.7-1231.8]
COVID-19 hospitalisation	ICS	No asthma	Adults	All	291,685	32,530,602	896.6 [893.4-899.9]	857.3 [854.1-860.4]
COVID-19 hospitalisation	ICS	No ICS	Adults	All	6,086	565,114	1077.0 [1050.0-1103.9]	1166.3 [1136.8-1195.8]
COVID-19 hospitalisation	ICS	Low ICS	Adults	All	18,689	1,451,443	1287.6 [1269.3-1306.0]	1175.4 [1158.2-1192.6]
COVID-19 hospitalisation	ICS	Medium ICS	Adults	All	10,907	519,294	2100.4 [2061.3-2139.4]	1729.2 [1693.8-1764.6]
COVID-19 hospitalisation	ICS	High ICS	Adults	All	3,696	136,080	2716.0 [2629.7-2802.4]	2074.4 [1999.4-2149.4]

COVID-19 hospitalisation	ICS	No asthma	Adults	Female	136,882	16,848,059	812.4 [808.2-816.7]	746.5 [742.5-750.5]
COVID-19 hospitalisation	ICS	No ICS	Adults	Female	3,467	327,992	1057.0 [1022.0-1092.0]	1100.6 [1063.7-1137.5]
COVID-19 hospitalisation	ICS	Low ICS	Adults	Female	10,984	871,384	1260.5 [1237.1-1283.9]	1141.3 [1119.4-1163.1]
COVID-19 hospitalisation	ICS	Medium ICS	Adults	Female	6,656	318,711	2088.4 [2038.8-2138.1]	1748.9 [1702.6-1795.2]
COVID-19 hospitalisation	ICS	High ICS	Adults	Female	2,091	82,058	2548.2 [2440.4-2656.0]	2008.3 [1911.9-2104.8]
COVID-19 hospitalisation	ICS	No asthma	Adults	Male	154,803	15,682,543	987.1 [982.2-992.0]	990.4 [985.5-995.4]
COVID-19 hospitalisation	ICS	No ICS	Adults	Male	2,619	237,122	1104.5 [1062.4-1146.6]	1275.2 [1225.4-1324.9]
COVID-19 hospitalisation	ICS	Low ICS	Adults	Male	7,705	580,059	1328.3 [1298.9-1357.8]	1244.6 [1216.0-1273.2]
COVID-19 hospitalisation	ICS	Medium ICS	Adults	Male	4,251	200,583	2119.3 [2056.3-2182.4]	1702.7 [1647.5-1758.0]
COVID-19 hospitalisation	ICS	High ICS	Adults	Male	1,605	54,022	2971.0 [2827.8-3114.2]	2162.8 [2043.8-2281.7]
COVID-19 hospitalisation	ICS	No asthma	18-39	All	30,035	10,625,503	282.7 [279.5-285.9]	284.6 [281.4-287.8]
COVID-19 hospitalisation	ICS	No ICS	18-39	All	819	221,307	370.1 [344.8-395.4]	382.3 [355.8-408.7]
COVID-19 hospitalisation	ICS	Low ICS	18-39	All	1,770	373,650	473.7 [451.7-495.7]	474.9 [452.8-497.0]
COVID-19 hospitalisation	ICS	Medium ICS	18-39	All	752	87,936	855.2 [794.3-916.0]	825.7 [765.9-885.4]
COVID-19 hospitalisation	ICS	High ICS	18-39	All	162	19,773	819.3 [693.7-944.9]	776.5 [655.9-897.2]
COVID-19 hospitalisation	ICS	No asthma	40-49	All	22,719	5,214,124	435.7 [430.1-441.4]	432.8 [427.2-438.4]
COVID-19 hospitalisation	ICS	No ICS	40-49	All	481	93,641	513.7 [467.9-559.5]	510.9 [465.2-556.6]
COVID-19 hospitalisation	ICS	Low ICS	40-49	All	1,787	233,875	764.1 [728.8-799.4]	752.0 [716.9-787.1]
COVID-19 hospitalisation	ICS	Medium ICS	40-49	All	1,000	77,770	1285.8 [1206.7-1365.0]	1268.1 [1188.6-1347.7]
COVID-19 hospitalisation	ICS	High ICS	40-49	All	293	17,826	1643.7 [1457.0-1830.3]	1576.9 [1393.6-1760.2]
COVID-19 hospitalisation	ICS	No asthma	50+	All	238,931	16,690,975	1431.5 [1425.8-1437.2]	1416.4 [1410.7-1422.0]
COVID-19 hospitalisation	ICS	No ICS	50+	All	4,786	250,166	1913.1 [1859.4-1966.8]	1958.4 [1902.7-2014.1]
COVID-19 hospitalisation	ICS	Low ICS	50+	All	15,132	843,918	1793.1 [1764.8-1821.4]	1825.0 [1795.7-1854.4]
COVID-19 hospitalisation	ICS	Medium ICS	50+	All	9,155	353,588	2589.2 [2536.8-2641.5]	2536.5 [2484.0-2588.9]
COVID-19 hospitalisation	ICS	High ICS	50+	All	3,241	98,481	3291.0 [3179.6-3402.4]	3174.9 [3063.5-3286.3]
COVID-19 hospitalisation	OCS	No asthma	Adults	All	291,685	32,530,602	896.6 [893.4-899.9]	857.3 [854.1-860.4]
COVID-19 hospitalisation	OCS	0 OCS	Adults	All	24,898	2,072,475	1201.4 [1186.5-1216.2]	1132.9 [1118.6-1147.1]
COVID-19 hospitalisation	OCS	1 OCS	Adults	All	5,457	307,486	1774.7 [1728.0-1821.4]	1570.6 [1527.2-1613.9]
COVID-19 hospitalisation	OCS	2+ OCS	Adults	All	9,023	291,970	3090.4 [3027.6-3153.2]	2369.0 [2312.3-2425.7]
COVID-19 hospitalisation	OCS	No asthma	Adults	Female	136,882	16,848,059	812.4 [808.2-816.7]	746.5 [742.5-750.5]
COVID-19 hospitalisation	OCS	0 OCS	Adults	Female	14,085	1,199,878	1173.9 [1154.6-1193.1]	1084.7 [1066.6-1102.8]

COVID-19 hospitalisation	OCS	1 OCS	Adults	Female	3,424	202,025	1694.8 [1638.6-1751.1]	1536.0 [1482.5-1589.6]
COVID-19 hospitalisation	OCS	2+ OCS	Adults	Female	5,689	198,242	2869.7 [2796.2-2943.2]	2296.7 [2228.7-2364.7]
COVID-19 hospitalisation	OCS	No asthma	Adults	Male	154,803	15,682,543	987.1 [982.2-992.0]	990.4 [985.5-995.4]
COVID-19 hospitalisation	OCS	0 OCS	Adults	Male	10,813	872,597	1239.2 [1216.0-1262.4]	1217.5 [1194.0-1241.0]
COVID-19 hospitalisation	OCS	1 OCS	Adults	Male	2,033	105,461	1927.7 [1844.7-2010.7]	1634.2 [1559.9-1708.6]
COVID-19 hospitalisation	OCS	2+ OCS	Adults	Male	3,334	93,728	3557.1 [3438.5-3675.7]	2480.3 [2378.5-2582.1]
COVID-19 hospitalisation	OCS	No asthma	18-39	All	30,035	10,625,503	282.7 [279.5-285.9]	284.6 [281.4-287.8]
COVID-19 hospitalisation	OCS	0 OCS	18-39	All	2,491	592,647	420.3 [403.8-436.8]	425.8 [409.1-442.6]
COVID-19 hospitalisation	OCS	1 OCS	18-39	All	527	68,195	772.8 [707.1-838.5]	760.3 [695.1-825.5]
COVID-19 hospitalisation	OCS	2+ OCS	18-39	All	485	41,824	1159.6 [1057.0-1262.2]	1114.5 [1014.2-1214.8]
COVID-19 hospitalisation	OCS	No asthma	40-49	All	22,719	5,214,124	435.7 [430.1-441.4]	432.8 [427.2-438.4]
COVID-19 hospitalisation	OCS	0 OCS	40-49	All	2,304	338,439	680.8 [653.1-708.5]	669.5 [642.1-697.0]
COVID-19 hospitalisation	OCS	1 OCS	40-49	All	572	48,571	1177.7 [1081.7-1273.6]	1162.8 [1066.8-1258.8]
COVID-19 hospitalisation	OCS	2+ OCS	40-49	All	685	36,102	1897.4 [1756.7-2038.1]	1878.5 [1736.0-2021.0]
COVID-19 hospitalisation	OCS	No asthma	50+	All	238,931	16,690,975	1431.5 [1425.8-1437.2]	1416.4 [1410.7-1422.0]
COVID-19 hospitalisation	OCS	0 OCS	50+	All	20,103	1,141,389	1761.3 [1737.1-1785.4]	1801.3 [1776.2-1826.4]
COVID-19 hospitalisation	OCS	1 OCS	50+	All	4,358	190,720	2285.0 [2218.0-2352.1]	2292.4 [2223.8-2361.1]
COVID-19 hospitalisation	OCS	2+ OCS	50+	All	7,853	214,044	3668.9 [3589.2-3748.5]	3436.1 [3358.1-3514.1]
COVID-19 hospitalisation	OCS	No asthma	Children	All	2,625	2,780,630	94.4 [90.8-98.0]	
COVID-19 hospitalisation	OCS	0 OCS	Children	All	254	197,867	128.4 [112.6-144.1]	
COVID-19 hospitalisation	OCS	1 OCS	Children	All	32	12,944	247.2 [161.7-332.8]	
COVID-19 hospitalisation	OCS	2+ OCS	Children	All	19	5,062	375.3 [206.9-543.8]	
COVID-19 hospitalisation	OCS	No asthma	Children	Female	1,543	1,375,527	112.2 [106.6-117.8]	
COVID-19 hospitalisation	OCS	0 OCS	Children	Female	142	87,276	162.7 [136.0-189.4]	
COVID-19 hospitalisation	OCS	1 OCS	Children	Female	20	5,755	347.5 [195.5-499.6]	
COVID-19 hospitalisation	OCS	2+ OCS	Children	Female	13	2,395	542.8 [248.5-837.1]	
COVID-19 hospitalisation	OCS	No asthma	Children	Male	1,082	1,405,103	77.0 [72.4-81.6]	
COVID-19 hospitalisation	OCS	0 OCS	Children	Male	112	110,591	101.3 [82.5-120.0]	
COVID-19 hospitalisation	OCS	1 OCS	Children	Male	12	7,189	166.9 [72.6-261.3]	
COVID-19 hospitalisation	OCS	2+ OCS	Children	Male	6	2,667	225.0 [45.2-404.8]	

Table S4 Hazard ratios of death involving COVID-19 and COVID-19 hospitalisation rates for different asthma group, stratified by age group, sex and vaccination status

Outcome	Age group	Stratification	Exposure	Exposure group	Model1	Model2	Model3	Model4
Death involving COVID-19	Adults	All	ICS	No ICS	1.42 [1.36-1.48]	1.35 [1.29-1.41]	1.12 [1.08-1.17]	1.12 [1.08-1.17]
Death involving COVID-19	Adults	All	ICS	Low ICS	1.08 [1.05-1.11]	1.04 [1.01-1.07]	1.01 [0.98-1.04]	1.02 [0.99-1.05]
Death involving COVID-19	Adults	All	ICS	Medium ICS	1.46 [1.40-1.51]	1.36 [1.32-1.41]	1.18 [1.14-1.22]	1.18 [1.14-1.23]
Death involving COVID-19	Adults	All	ICS	High ICS	1.89 [1.78-2.00]	1.75 [1.65-1.86]	1.34 [1.26-1.42]	1.36 [1.28-1.44]
Death involving COVID-19	Adults	Male	ICS	No ICS	1.35 [1.27-1.44]	1.28 [1.20-1.36]	1.06 [1.00-1.13]	1.06 [1.00-1.13]
Death involving COVID-19	Adults	Male	ICS	Low ICS	0.99 [0.95-1.04]	0.96 [0.92-1.00]	0.92 [0.89-0.96]	0.94 [0.90-0.98]
Death involving COVID-19	Adults	Male	ICS	Medium ICS	1.27 [1.20-1.34]	1.20 [1.14-1.26]	1.02 [0.96-1.07]	1.03 [0.97-1.08]
Death involving COVID-19	Adults	Male	ICS	High ICS	1.73 [1.59-1.88]	1.59 [1.47-1.73]	1.24 [1.14-1.35]	1.26 [1.16-1.37]
Death involving COVID-19	Adults	Female	ICS	No ICS	1.49 [1.41-1.59]	1.43 [1.34-1.52]	1.18 [1.11-1.25]	1.17 [1.11-1.25]
Death involving COVID-19	Adults	Female	ICS	Low ICS	1.17 [1.13-1.21]	1.12 [1.07-1.16]	1.08 [1.04-1.12]	1.09 [1.04-1.13]
Death involving COVID-19	Adults	Female	ICS	Medium ICS	1.64 [1.57-1.72]	1.53 [1.46-1.60]	1.33 [1.26-1.39]	1.33 [1.27-1.40]
Death involving COVID-19	Adults	Female	ICS	High ICS	2.06 [1.90-2.23]	1.92 [1.77-2.08]	1.43 [1.32-1.55]	1.45 [1.33-1.57]
Death involving COVID-19	Adults	18-39	ICS	No ICS	1.72 [1.14-2.61]	1.72 [1.14-2.61]	1.58 [1.04-2.39]	1.61 [1.06-2.45]
Death involving COVID-19	Adults	18-39	ICS	Low ICS	1.94 [1.45-2.61]	1.94 [1.45-2.61]	1.41 [1.03-1.93]	1.45 [1.05-1.98]
Death involving COVID-19	Adults	18-39	ICS	Medium ICS	5.04 [3.52-7.23]	5.04 [3.51-7.23]	2.84 [1.90-4.23]	2.95 [1.98-4.39]
Death involving COVID-19	Adults	18-39	ICS	High ICS	5.88 [2.93-11.80]	5.79 [2.88-11.63]	2.91 [1.40-6.05]	3.11 [1.49-6.45]
Death involving COVID-19	Adults	40-49	ICS	No ICS	1.32 [0.95-1.83]	1.36 [0.97-1.89]	1.16 [0.83-1.62]	1.17 [0.84-1.63]
Death involving COVID-19	Adults	40-49	ICS	Low ICS	2.14 [1.81-2.54]	2.13 [1.80-2.53]	1.51 [1.26-1.82]	1.54 [1.28-1.84]
Death involving COVID-19	Adults	40-49	ICS	Medium ICS	3.62 [2.91-4.52]	3.59 [2.87-4.48]	1.89 [1.49-2.41]	1.95 [1.53-2.47]
Death involving COVID-19	Adults	40-49	ICS	High ICS	5.38 [3.68-7.87]	5.09 [3.47-7.45]	2.22 [1.49-3.30]	2.22 [1.49-3.30]
Death involving COVID-19	Adults	50+	ICS	No ICS	1.42 [1.36-1.49]	1.35 [1.29-1.41]	1.12 [1.07-1.17]	1.12 [1.07-1.17]
Death involving COVID-19	Adults	50+	ICS	Low ICS	1.06 [1.03-1.09]	1.02 [0.99-1.05]	0.99 [0.96-1.02]	1.00 [0.97-1.03]
Death involving COVID-19	Adults	50+	ICS	Medium ICS	1.42 [1.37-1.47]	1.33 [1.29-1.38]	1.15 [1.11-1.20]	1.16 [1.12-1.20]
Death involving COVID-19	Adults	50+	ICS	High ICS	1.85 [1.74-1.96]	1.72 [1.62-1.82]	1.31 [1.24-1.40]	1.33 [1.26-1.42]
Death involving COVID-19	Adults	Vaccinated	ICS	No ICS	1.51 [1.31-1.73]	1.45 [1.26-1.67]	1.14 [0.99-1.31]	
Death involving COVID-19	Adults	Vaccinated	ICS	Low ICS	1.08 [0.99-1.18]	1.05 [0.96-1.14]	0.95 [0.87-1.04]	
Death involving COVID-19	Adults	Vaccinated	ICS	Medium ICS	1.64 [1.47-1.82]	1.56 [1.40-1.73]	1.24 [1.11-1.38]	
Death involving COVID-19	Adults	Vaccinated	ICS	High ICS	1.92 [1.60-2.30]	1.78 [1.49-2.13]	1.21 [1.00-1.45]	

Death involving COVID-19	Adults	Unvaccinated	ICS	No ICS	1.41 [1.34-1.47]	1.34 [1.28-1.40]	1.12 [1.07-1.18]	
Death involving COVID-19	Adults	Unvaccinated	ICS	Low ICS	1.09 [1.06-1.12]	1.05 [1.02-1.08]	1.02 [0.99-1.05]	
Death involving COVID-19	Adults	Unvaccinated	ICS	Medium ICS	1.45 [1.40-1.51]	1.36 [1.31-1.41]	1.18 [1.13-1.22]	
Death involving COVID-19	Adults	Unvaccinated	ICS	High ICS	1.92 [1.81-2.04]	1.78 [1.68-1.89]	1.37 [1.29-1.46]	
Death involving COVID-19	Adults	All	OCS	0 OCS	1.09 [1.06-1.12]	1.04 [1.02-1.07]	1.09 [1.06-1.12]	1.10 [1.07-1.12]
Death involving COVID-19	Adults	All	OCS	1 OCS	1.39 [1.32-1.46]	1.28 [1.22-1.35]	1.19 [1.13-1.25]	1.20 [1.14-1.26]
Death involving COVID-19	Adults	All	OCS	2+ OCS	2.09 [2.01-2.16]	1.95 [1.88-2.02]	1.59 [1.53-1.65]	1.60 [1.55-1.67]
Death involving COVID-19	Adults	Male	OCS	0 OCS	1.03 [1.00-1.07]	0.99 [0.96-1.03]	1.03 [0.99-1.06]	1.04 [1.00-1.07]
Death involving COVID-19	Adults	Male	OCS	1 OCS	1.24 [1.15-1.34]	1.14 [1.06-1.24]	1.04 [0.96-1.13]	1.05 [0.97-1.14]
Death involving COVID-19	Adults	Male	OCS	2+ OCS	1.78 [1.68-1.89]	1.66 [1.57-1.76]	1.32 [1.24-1.39]	1.33 [1.25-1.41]
Death involving COVID-19	Adults	Female	OCS	0 OCS	1.15 [1.11-1.19]	1.10 [1.06-1.13]	1.15 [1.11-1.19]	1.15 [1.12-1.19]
Death involving COVID-19	Adults	Female	OCS	1 OCS	1.52 [1.42-1.62]	1.41 [1.32-1.51]	1.32 [1.23-1.41]	1.33 [1.24-1.42]
Death involving COVID-19	Adults	Female	OCS	2+ OCS	2.37 [2.26-2.48]	2.21 [2.11-2.32]	1.82 [1.73-1.91]	1.83 [1.75-1.93]
Death involving COVID-19	Adults	18-39	OCS	0 OCS	1.96 [1.54-2.49]	1.98 [1.56-2.52]	1.83 [1.43-2.34]	1.88 [1.47-2.41]
Death involving COVID-19	Adults	18-39	OCS	1 OCS	2.00 [1.03-3.86]	1.87 [0.97-3.61]	1.98 [1.02-3.82]	2.03 [1.05-3.92]
Death involving COVID-19	Adults	18-39	OCS	2+ OCS	9.61 [6.49-14.24]	9.04 [6.10-13.41]	7.36 [4.93-10.99]	7.72 [5.17-11.53]
Death involving COVID-19	Adults	40-49	OCS	0 OCS	1.78 [1.53-2.08]	1.82 [1.56-2.12]	1.74 [1.48-2.03]	1.78 [1.52-2.08]
Death involving COVID-19	Adults	40-49	OCS	1 OCS	3.49 [2.62-4.66]	3.24 [2.43-4.33]	3.02 [2.26-4.03]	3.00 [2.24-4.02]
Death involving COVID-19	Adults	40-49	OCS	2+ OCS	6.82 [5.32-8.73]	6.28 [4.90-8.05]	4.56 [3.52-5.91]	4.61 [3.56-5.98]
Death involving COVID-19	Adults	50+	OCS	0 OCS	1.07 [1.05-1.10]	1.03 [1.00-1.05]	1.07 [1.05-1.10]	1.08 [1.05-1.11]
Death involving COVID-19	Adults	50+	OCS	1 OCS	1.36 [1.29-1.43]	1.26 [1.20-1.33]	1.16 [1.10-1.22]	1.17 [1.11-1.24]
Death involving COVID-19	Adults	50+	OCS	2+ OCS	2.04 [1.96-2.12]	1.90 [1.84-1.98]	1.55 [1.49-1.61]	1.57 [1.51-1.63]
Death involving COVID-19	Adults	Vaccinated	OCS	0 OCS	1.13 [1.05-1.22]	1.10 [1.02-1.18]	1.12 [1.04-1.21]	
Death involving COVID-19	Adults	Vaccinated	OCS	1 OCS	1.36 [1.15-1.60]	1.27 [1.08-1.49]	1.08 [0.92-1.28]	
Death involving COVID-19	Adults	Vaccinated	OCS	2+ OCS	2.27 [2.03-2.54]	2.13 [1.91-2.38]	1.60 [1.43-1.79]	
Death involving COVID-19	Adults	Unvaccinated	OCS	0 OCS	1.09 [1.06-1.12]	1.05 [1.02-1.07]	1.09 [1.07-1.12]	
Death involving COVID-19	Adults	Unvaccinated	OCS	1 OCS	1.40 [1.33-1.48]	1.30 [1.23-1.37]	1.21 [1.15-1.28]	
Death involving COVID-19	Adults	Unvaccinated	OCS	2+ OCS	2.10 [2.02-2.18]	1.96 [1.88-2.03]	1.60 [1.54-1.67]	
COVID-19 hospitalisation	Adults	All	ICS	No ICS	1.41 [1.37-1.44]	1.35 [1.32-1.39]	1.15 [1.13-1.18]	1.17 [1.14-1.20]
COVID-19 hospitalisation	Adults	All	ICS	Low ICS	1.39 [1.37-1.41]	1.34 [1.32-1.36]	1.26 [1.25-1.28]	1.28 [1.27-1.30]
COVID-19 hospitalisation	Adults	All	ICS	Medium ICS	1.96 [1.92-1.99]	1.83 [1.80-1.87]	1.51 [1.48-1.54]	1.53 [1.50-1.56]
COVID-19 hospitalisation	Adults	All	ICS	High ICS	2.41 [2.33-2.49]	2.17 [2.11-2.25]	1.49 [1.44-1.54]	1.51 [1.46-1.56]
COVID-19 hospitalisation	Adults	Male	ICS	No ICS	1.32 [1.27-1.37]	1.26 [1.22-1.31]	1.08 [1.04-1.12]	1.09 [1.05-1.13]

COVID-19 hospitalisation	Adults	Male	ICS	Low ICS	1.24 [1.22-1.27]	1.21 [1.18-1.24]	1.17 [1.14-1.19]	1.18 [1.16-1.21]
COVID-19 hospitalisation	Adults	Male	ICS	Medium ICS	1.67 [1.62-1.73]	1.57 [1.52-1.61]	1.31 [1.27-1.35]	1.33 [1.29-1.37]
COVID-19 hospitalisation	Adults	Male	ICS	High ICS	2.16 [2.06-2.27]	1.97 [1.88-2.07]	1.42 [1.35-1.49]	1.44 [1.37-1.51]
COVID-19 hospitalisation	Adults	Female	ICS	No ICS	1.49 [1.44-1.54]	1.43 [1.38-1.48]	1.22 [1.18-1.26]	1.23 [1.19-1.27]
COVID-19 hospitalisation	Adults	Female	ICS	Low ICS	1.51 [1.48-1.54]	1.45 [1.42-1.47]	1.34 [1.32-1.37]	1.36 [1.34-1.39]
COVID-19 hospitalisation	Adults	Female	ICS	Medium ICS	2.20 [2.15-2.26]	2.06 [2.01-2.11]	1.67 [1.63-1.71]	1.70 [1.66-1.74]
COVID-19 hospitalisation	Adults	Female	ICS	High ICS	2.64 [2.53-2.76]	2.36 [2.26-2.46]	1.54 [1.47-1.60]	1.55 [1.49-1.62]
COVID-19 hospitalisation	Adults	18-39	ICS	No ICS	1.27 [1.18-1.36]	1.27 [1.19-1.37]	1.20 [1.12-1.29]	1.25 [1.17-1.35]
COVID-19 hospitalisation	Adults	18-39	ICS	Low ICS	1.59 [1.52-1.67]	1.58 [1.51-1.66]	1.39 [1.32-1.46]	1.49 [1.41-1.56]
COVID-19 hospitalisation	Adults	18-39	ICS	Medium ICS	2.75 [2.56-2.96]	2.77 [2.58-2.98]	2.08 [1.92-2.24]	2.26 [2.09-2.44]
COVID-19 hospitalisation	Adults	18-39	ICS	High ICS	2.78 [2.38-3.24]	2.69 [2.31-3.14]	1.79 [1.52-2.09]	1.92 [1.64-2.25]
COVID-19 hospitalisation	Adults	40-49	ICS	No ICS	1.18 [1.08-1.29]	1.22 [1.11-1.33]	1.11 [1.01-1.22]	1.14 [1.04-1.24]
COVID-19 hospitalisation	Adults	40-49	ICS	Low ICS	1.78 [1.70-1.87]	1.82 [1.73-1.91]	1.52 [1.45-1.60]	1.56 [1.49-1.65]
COVID-19 hospitalisation	Adults	40-49	ICS	Medium ICS	3.00 [2.81-3.19]	3.02 [2.83-3.21]	2.13 [1.99-2.28]	2.21 [2.06-2.36]
COVID-19 hospitalisation	Adults	40-49	ICS	High ICS	3.83 [3.42-4.30]	3.73 [3.32-4.19]	2.39 [2.12-2.70]	2.49 [2.21-2.81]
COVID-19 hospitalisation	Adults	50+	ICS	No ICS	1.46 [1.42-1.50]	1.38 [1.34-1.42]	1.15 [1.11-1.18]	1.15 [1.12-1.18]
COVID-19 hospitalisation	Adults	50+	ICS	Low ICS	1.33 [1.31-1.35]	1.27 [1.25-1.30]	1.22 [1.20-1.24]	1.23 [1.21-1.25]
COVID-19 hospitalisation	Adults	50+	ICS	Medium ICS	1.84 [1.80-1.88]	1.71 [1.67-1.74]	1.42 [1.39-1.45]	1.43 [1.40-1.47]
COVID-19 hospitalisation	Adults	50+	ICS	High ICS	2.30 [2.23-2.38]	2.06 [1.99-2.14]	1.42 [1.37-1.48]	1.43 [1.38-1.49]
COVID-19 hospitalisation	Adults	Vaccinated	ICS	No ICS	1.47 [1.36-1.60]	1.43 [1.32-1.54]	1.14 [1.05-1.24]	
COVID-19 hospitalisation	Adults	Vaccinated	ICS	Low ICS	1.51 [1.44-1.57]	1.45 [1.39-1.52]	1.32 [1.26-1.38]	
COVID-19 hospitalisation	Adults	Vaccinated	ICS	Medium ICS	2.20 [2.08-2.32]	2.06 [1.95-2.18]	1.56 [1.48-1.66]	
COVID-19 hospitalisation	Adults	Vaccinated	ICS	High ICS	2.86 [2.61-3.14]	2.53 [2.31-2.77]	1.53 [1.40-1.68]	
COVID-19 hospitalisation	Adults	Unvaccinated	ICS	No ICS	1.42 [1.38-1.46]	1.36 [1.32-1.40]	1.17 [1.14-1.20]	
COVID-19 hospitalisation	Adults	Unvaccinated	ICS	Low ICS	1.40 [1.38-1.42]	1.35 [1.32-1.37]	1.28 [1.26-1.30]	
COVID-19 hospitalisation	Adults	Unvaccinated	ICS	Medium ICS	1.96 [1.92-2.00]	1.84 [1.80-1.88]	1.53 [1.50-1.56]	
COVID-19 hospitalisation	Adults	Unvaccinated	ICS	High ICS	2.39 [2.31-2.47]	2.17 [2.09-2.24]	1.51 [1.45-1.56]	
COVID-19 hospitalisation	Adults	All	OCS	0 OCS	1.34 [1.32-1.36]	1.29 [1.28-1.31]	1.31 [1.29-1.33]	1.33 [1.31-1.35]
COVID-19 hospitalisation	Adults	All	OCS	1 OCS	1.82 [1.77-1.87]	1.69 [1.65-1.74]	1.56 [1.52-1.60]	1.59 [1.55-1.63]
COVID-19 hospitalisation	Adults	All	OCS	2+ OCS	2.70 [2.65-2.76]	2.49 [2.44-2.54]	1.92 [1.87-1.96]	1.94 [1.90-1.99]
COVID-19 hospitalisation	Adults	Male	OCS	0 OCS	1.23 [1.21-1.26]	1.19 [1.17-1.22]	1.20 [1.18-1.23]	1.22 [1.20-1.24]
COVID-19 hospitalisation	Adults	Male	OCS	1 OCS	1.60 [1.53-1.67]	1.49 [1.42-1.55]	1.36 [1.30-1.42]	1.39 [1.33-1.45]
COVID-19 hospitalisation	Adults	Male	OCS	2+ OCS	2.36 [2.28-2.44]	2.16 [2.08-2.23]	1.65 [1.60-1.71]	1.67 [1.62-1.73]

COVID-19 hospitalisation	Adults	Female	OCS	0 OCS	1.44 [1.42-1.47]	1.38 [1.36-1.41]	1.40 [1.38-1.43]	1.43 [1.40-1.45]
COVID-19 hospitalisation	Adults	Female	OCS	1 OCS	2.00 [1.93-2.07]	1.84 [1.78-1.90]	1.70 [1.64-1.76]	1.73 [1.67-1.79]
COVID-19 hospitalisation	Adults	Female	OCS	2+ OCS	2.97 [2.89-3.05]	2.73 [2.66-2.80]	2.08 [2.03-2.14]	2.11 [2.06-2.17]
COVID-19 hospitalisation	Adults	18-39	OCS	0 OCS	1.44 [1.38-1.50]	1.44 [1.38-1.50]	1.46 [1.40-1.52]	1.56 [1.49-1.62]
COVID-19 hospitalisation	Adults	18-39	OCS	1 OCS	2.45 [2.24-2.67]	2.33 [2.14-2.54]	2.33 [2.14-2.54]	2.54 [2.33-2.77]
COVID-19 hospitalisation	Adults	18-39	OCS	2+ OCS	3.56 [3.26-3.90]	3.48 [3.18-3.80]	2.93 [2.68-3.21]	3.23 [2.95-3.54]
COVID-19 hospitalisation	Adults	40-49	OCS	0 OCS	1.57 [1.51-1.64]	1.62 [1.56-1.70]	1.58 [1.52-1.65]	1.63 [1.56-1.70]
COVID-19 hospitalisation	Adults	40-49	OCS	1 OCS	2.72 [2.50-2.96]	2.64 [2.43-2.87]	2.51 [2.31-2.73]	2.60 [2.39-2.83]
COVID-19 hospitalisation	Adults	40-49	OCS	2+ OCS	4.71 [4.37-5.09]	4.50 [4.17-4.86]	3.62 [3.34-3.91]	3.75 [3.46-4.06]
COVID-19 hospitalisation	Adults	50+	OCS	0 OCS	1.31 [1.29-1.33]	1.25 [1.23-1.26]	1.26 [1.25-1.28]	1.27 [1.25-1.29]
COVID-19 hospitalisation	Adults	50+	OCS	1 OCS	1.69 [1.64-1.75]	1.56 [1.52-1.61]	1.43 [1.39-1.47]	1.44 [1.40-1.49]
COVID-19 hospitalisation	Adults	50+	OCS	2+ OCS	2.56 [2.50-2.62]	2.35 [2.29-2.40]	1.79 [1.75-1.84]	1.81 [1.77-1.85]
COVID-19 hospitalisation	Adults	Vaccinated	OCS	0 OCS	1.39 [1.34-1.45]	1.35 [1.29-1.40]	1.36 [1.31-1.41]	
COVID-19 hospitalisation	Adults	Vaccinated	OCS	1 OCS	2.10 [1.95-2.27]	1.93 [1.79-2.09]	1.72 [1.59-1.86]	
COVID-19 hospitalisation	Adults	Vaccinated	OCS	2+ OCS	3.34 [3.15-3.54]	3.03 [2.86-3.21]	2.17 [2.05-2.31]	
COVID-19 hospitalisation	Adults	Unvaccinated	OCS	0 OCS	1.36 [1.34-1.38]	1.31 [1.29-1.32]	1.32 [1.30-1.34]	
COVID-19 hospitalisation	Adults	Unvaccinated	OCS	1 OCS	1.83 [1.78-1.88]	1.69 [1.65-1.74]	1.57 [1.52-1.61]	
COVID-19 hospitalisation	Adults	Unvaccinated	OCS	2+ OCS	2.67 [2.61-2.73]	2.47 [2.41-2.52]	1.91 [1.87-1.95]	
COVID-19 hospitalisation	Children	All	OCS	0 OCS	1.39 [1.22-1.58]	1.35 [1.19-1.54]	1.44 [1.27-1.64]	
COVID-19 hospitalisation	Children	All	OCS	1 OCS	2.72 [1.92-3.86]	2.49 [1.76-3.53]	2.58 [1.82-3.66]	
COVID-19 hospitalisation	Children	All	OCS	2+ OCS	4.08 [2.60-6.41]	3.78 [2.40-5.93]	3.79 [2.41-5.95]	
COVID-19 hospitalisation	Children	Male	OCS	0 OCS	1.33 [1.09-1.61]	1.26 [1.03-1.53]	1.35 [1.11-1.64]	
COVID-19 hospitalisation	Children	Male	OCS	1 OCS	2.27 [1.28-4.01]	2.01 [1.14-3.55]	2.13 [1.20-3.76]	
COVID-19 hospitalisation	Children	Male	OCS	2+ OCS	3.10 [1.39-6.93]	2.74 [1.23-6.12]	2.77 [1.24-6.18]	
COVID-19 hospitalisation	Children	Female	OCS	0 OCS	1.44 [1.21-1.71]	1.41 [1.19-1.68]	1.50 [1.27-1.79]	

COVID-19 hospitalisation	Children	Female	OCS	1 OCS	3.09 [1.99-4.80]	2.86 [1.84-4.44]	2.93 [1.88-4.55]
COVID-19 hospitalisation	Children	Female	OCS	2+ OCS	4.78 [2.77-8.24]	4.46 [2.58-7.70]	4.47 [2.59-7.71]

Note: Model 1 is adjusted for age and sex – sex is not included when estimates are stratified by sex. Model 2 includes the Model 1 adjustments and further adjusts for socio-demographic factors, including region, ethnicity and quintile of Index of Multiple Deprivation. Model 3 includes the Model 2 adjustments and further adjusts for comorbidities, hospitalisation in the previous year for any other reason than asthma, and OCS use – comorbidities were not included for children and OCS use was not included when OCS was the exposure. Model 4 includes the Model 3 adjustments and further adjusts for vaccine status as a time varying covariate – vaccine status was not included when estimates are stratified by vaccine status or for children.
